# Audit of Antimicrobial Prescribing Trends in 1447 Outpatient Wound Assessments: Baseline Rates and Impact of Bacterial Fluorescence Imaging

**DOI:** 10.3390/diagnostics14182034

**Published:** 2024-09-13

**Authors:** Nancy Trafelet, Scott Johnson, Jill Schroder, Thomas E. Serena

**Affiliations:** 1 SerenaGroup® Inc., 125 Cambridge Park Drive Suite 301, Cambridge, MA 02140, USA; ntrafelet@serenagroups.com (N.T.); jschroder@serenagroups.com (J.S.); 2 Ascension Via Christi Wound Center, Wichita, KS 67214, USA; scott.johnson21@ascension.org

**Keywords:** fluorescence imaging, antibiotic stewardship, antimicrobials, chronic wounds, bacterial burden, biofilm

## Abstract

**Background/Objectives:** In the field of wound care, the prescription of antibiotics and antimicrobials is haphazard and irrational, which has led to unchecked overprescribing. Recent Joint Commission guidelines mandate that hospital outpatient clinics develop and implement antimicrobial stewardship programs (ASPs). Yet few ASPs exist in wound clinics across the United States (US). Understanding baseline prescribing practices and rates in the US is a critical first step toward rational antimicrobial use and effective ASPs. **Methods:** This prospective study was conducted across eight outpatient wound clinics from January–December 2022. Data from consecutive patients attending single-time-point initial visits were recorded, including clinical findings, antimicrobial prescribing trends, and sampling practices. **Results:** A total of 1438 wounds were included; 964 were assessed by clinical examination (standard of care, SoC), and 474 by clinical examination plus fluorescence imaging. SoC patients were prescribed more concurrent medications on average than fluorescence patients (1.4 vs. 1 per patient). Prescriptions were preferentially topical in the fluorescence group (92% vs. 64%, *p* > 0.0001), and systemic antibiotics represented 36% of the single items prescribed under SoC (vs. 8% in fluorescence group *p* < 0.0001). **Conclusions:** Fluorescence imaging provided objective and actionable information at the bedside, which led to a decrease in the use of antibiotics. Real-time diagnostic technologies are essential in establishing a meaningful ASP.

## 1. Introduction

According to the Centers for Disease Control (CDC), approximately 30% of antibiotic prescriptions are unnecessary [1]. Skin and soft tissue infections account for a significant number of unsupported prescriptions, over half of which are made without clear medical necessity [2]. This practice contributes to the growing problem of antibiotic resistance [3]. Bacterial levels above 10^5^ CFU/gram, referred to as the chronic inhibitory bacterial load (CIBL), impede wound healing and may lead to system infection, sepsis, amputation, and death [4,5,6]. Unfortunately, clinical signs and symptoms of the CIBL and worse conditions are insensitive [7], and the semi-quantitative cultures used in most hospital laboratories are inaccurate [8]. Establishing a meaningful antimicrobial stewardship program (ASP) requires accurate point-of-care identification of bacteria to guide the treatment plan [9].

Diagnostic uncertainty in the field of wound care plays a major role in antibiotic misuse. The European Wound Management Association (EWMA) identified it as a key reason for high antibiotic prescribing rates [10]. Persistent reliance solely on clinical examination and semi-quantitative cultures has led to haphazard antibiotic prescribing [11]. Antibiotic stewardship programs (ASPs) play a crucial role in promoting the appropriate and responsible use of antibiotics. ASPs aim to optimize antibiotic prescribing practices in healthcare settings to ultimately improve patient outcomes, prevent the development of antibiotic resistance, and reduce healthcare costs [12].

Research in hard-to-heal wounds suggests that local measures, such as repeated debridement and topical antiseptics, may be sufficient to treat bacteria and biofilms localized to the wound bed. Systemic antibiotics should be reserved for infections that spread beyond the wound or for patients with systemic signs of infection [13]. In the absence of point-of-care diagnostics and ASPs, patients with hard-to-heal wounds continue to receive a significantly higher number of systemic antibiotic prescriptions compared to age- and gender-matched individuals without open wounds [14]. Prescribing rates are estimated to be between 47 and 79% in long-term care [15], and between 53 and 71% in outpatient chronic wound clinics [16]. These figures likely underestimate antibiotic prescription according to studies conducted in long-term care and skilled nursing facilities [17,18]. These data suggest that clinicians are uncomfortable using local therapies in the treatment of hard-to-heal wounds.

In a 350-patient clinical trial, fluorescence imaging (MolecuLight^®^, Toronto, ON, Canada) accurately detected the CIBL (>10^4^ CFU/gr) in real time [7]. The point-of-care device illuminates the wound with a violet light (405 nm). Bacterial porphyrins and pyoverdines in turn emit a fluorescence signal: red for most bacterial species, and cyan for *Pseudomonas* [19]. The trial findings showed that these fluorescence signals were highly predictive for bacterial loads at and above the CIBL level. The positive predictive value (PPV) ranged between 93 and 100% [7]. In addition, fluorescence imaging changed the treatment plan in 42% of cases [7]. Subsequent studies demonstrated that fluorescence imaging increased the detection of clinically significant levels of bacteria by up to 11-fold in terms of clinical signs and symptoms alone [20]. SerenaGroup^®^ Inc. (Cambridge, MA, USA) developed an ASP using fluorescence imaging in accordance with the Joint Commission guidelines [21] and published the ASP in a peer-reviewed journal [7].

In 2022, not all the wound centers included in the ASP had obtained fluorescence imaging devices. ASP data were collected from seven outpatient wound care centers, geographically spread across the United States. A comparison of topical antiseptic and systemic antibiotic use between centers using fluorescence imaging and those without the technology was planned.

## 2. Methods

This prospective multicenter study was conducted in accordance with the Joint Commission mandate to establish an ASP outpatient program and publish the findings. SerenaGroup^®^ Inc. (SGI) initiated an ASP in 2022. A policy for an ASP was developed, approved by hospital compliance, and included in each of the hospital’s policy and procedure manuals. Deidentified data were then collected from 7 specialized outpatient wound care centers across the US. Information was uniformly collected from these sites via a questionnaire that was designed by an ASP committee.

ASP data were collected by 23 different clinicians (16 Medical Doctors (MDs), 6 Doctors of Podiatric Medicine (DPMs), and 1 Nurse Practitioner (NP)) immediately after each patient encounter and transcribed by a single abstractor (NP) to a centralized, password-protected database. No protected health information (PHI) was included on the questionnaire, and the abstractor ensured the confidentiality of the information. Data recorded in questionnaire format included the following: (1) clinical signs and symptoms of wound infection’s presence (or absence); (2) bacterial load FL-imaging findings, if performed; (3) the prescription of any topical or systemic antimicrobials (including antibiotics); and (4) culture sampling and the method used, if performed. The data were separated into two groups: of note, wound standard of care (SoC), or SoC in combination with fluorescence imaging.

***Inclusion/exclusion criteria:*** Consecutive patients who attended for a first-time wound evaluation to one of the 7 sites between 1 January 2022 and 31 December 2022 were included. These were hospital outpatient wound care centers in Huntington, WV; Akron, OH; Bangor, ME; Seville, MI; Chesterfield, MI; Omaha, NB; and Montgomery, AL. Follow-up visits were excluded. No further exclusion/inclusion criteria were applied.

***Clinical assessment:*** Clinical evaluation for all clinicians followed the *SerenaGroup Practice of Wound Care Guidelines & Evidence-Based Wound Care Practice Guidelines criteria* (2021). This document is based on national and international guidelines for wound management [22,23,24,25,26]. The decision on whether to collect a sample for microbiology was made at the discretion of the treating clinician.

After the collection was deemed complete, the centralized dataset underwent a blind audit by two members of the ASP Committee. Checks were performed for consistency and accuracy.

***Statistical analysis:*** Statistical analysis was performed using GraphPad PRISM, Version 9.2.0, 2021. The proportions and absolute numbers found were compared, as appropriate, between the two groups (SoC vs. Fluorescence). Statistical significance was determined using Fisher’s exact test when comparing decisions leading to prescriptions, the number of prescriptions made, and topical versus systemic medication use. A two-tailed Fisher’s exact test was used to compare the use of the different topical and systemic medications. To further analyze the different types of medications used in both groups, the Chi-Square test was applied. An alpha value was considered significant when *p* < 0.05.

## 3. Results

Prescription practices from 1447 individual, consecutive wound assessments were included for initial analysis with no exclusion criteria. Nine wounds were excluded because they had healed at the time of assessment, leaving 1438 wounds suitable for analysis. Of those, 964 were assessed by clinical examination alone (SoC), and 474 by clinical examination in combination with fluorescence imaging. Data from these two groups were analyzed and compared.

### 3.1. Overview of Prescribing Practice

Antimicrobials of any kind were used in 63% of all study patients, where 487 belonged to the SoC group and 414 belonged to the fluorescence group. A total of 1061 individual items were prescribed overall. These included antimicrobial dressings (38%), topical antibiotics (27%), oral antibiotics (24%), and topical antiseptics (9.5%). Less than 2% of patients received IV antibiotics, advanced wound care (negative pressure wound therapy) (NPWT), or were referred to an infectious disease (ID) specialist.

A comparison of the total number of medications of any kind (topical and/or systemic) prescribed between the groups demonstrated a statistically significant difference, where there were proportionally more prescriptions made in the fluorescence group (87.3%) compared to the SoC group (50.5%) *p* < 0.0001. However, each of the prescribed patients in the SoC arm was given more concurrent medications during that initial visit than those in the fluorescence group. In the SoC group, 487 prescribed patients received 663 single items (e.g., medications/dressings, etc.), which averages 1.4 items per patient. In the fluorescence group, there was an average of 1 item prescribed per patient (425 single items for 414 patients).

There was a marked contrast between the groups in the type of medications that were selected. Those exposed to fluorescence imaging were predominantly prescribed topical medications and dressings, while in the standard-of-care (SoC) group, there seemed to be a distinct preference for systemic medications.

### 3.2. Type of Medications Prescribed per Group

**Figure 1:**
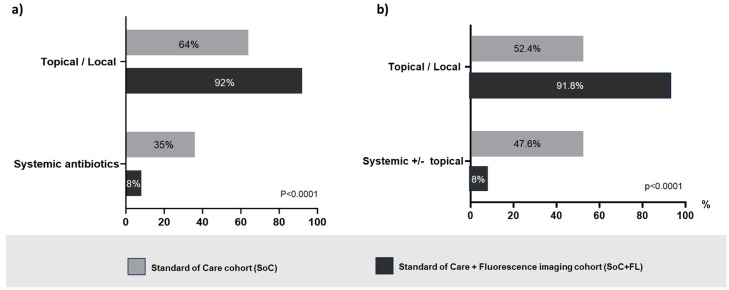
(**a**) Comparative proportional distribution of the treatment selections per single item prescribed (systemic antibiotics versus topical antibiotics and antiseptics, and/or antimicrobial dressings) showing a statistically significant preference for systemic antibiotics in the standard-of-care (SoC group, grey bars) group. Conversely, patients assessed by the standard of care in conjunction with fluorescence imaging (FL group, black bars) were prescribed significantly more topical/local measures and medications. (**b**) Comparative proportional distribution of treatment selections per patient (systemic antibiotics alone or in combination with topical versus only topical/local treatments) reveals a preference for systemic antibiotics in the SoC (grey bars) group (*p* < 0.001). Conversely, wound assessments involving fluorescence imaging (black bars) resulted in more topical/local measures and medications.

The comparison of topical local versus systemic medications used by clinicians in each group revealed a statistically significant preference for local interventions in fluorescence imaging. Conversely, in the SoC arm, systemic antibiotics comprised a sizable portion of the prescriptions made. On a per-item basis, systemic antibiotics constituted 35% (*n* = 224) of single items prescribed in the SoC group, whereas the fluoresce group exhibited only an 8% (*n* = 34) systemic antibiotic usage rate (*p* < 0.0001) (Figure 1a).

**Figure 2:**
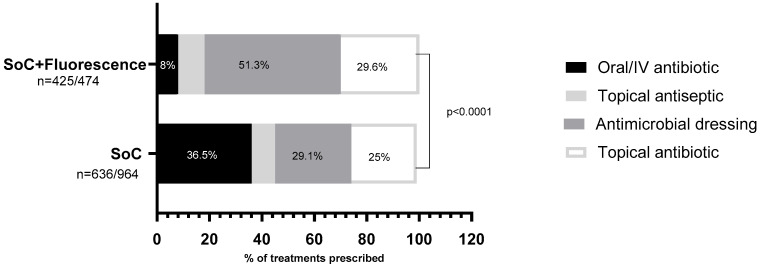
Distribution of the types of treatment selected in each one of the study groups. A statistically significant preference for systemic antibiotics is evidenced (in black) in the SoC (standard-of-care) group, representing a third of the chosen prescriptions; however, this type of medication is used in only 8% of the patients assessed in conjunction with fluorescence imaging. In this group, the preferential treatment was antimicrobial dressings, underscoring a marked preference towards local treatment.

On a per-patient basis, systemic antibiotics were used alone or in combination with topicals in 47.6% (*n* = 232) of SoC patients versus 8.2% (*n* = 34) of patients in the fluorescence group (*p* < 0.0001) (Figure 1b). Figure 2 illustrates the details of prescribed medications among patients who received prescriptions (SoC *n* = 487, fluorescence group *n* = 414). Without considering those patients requiring advanced therapies and IV antibiotics, who likely were more severely compromised, the prescription of topical agents including antimicrobial dressings, antibiotics, and antiseptics was 44% greater in the fluorescence group (64%) than in the SoC group (92%).

A proportion of the patients had tissue sampling performed for microbiological analysis of their wounds at this first visit. All samples were collected via swabbing and sent for PCR testing. While awaiting these confirmatory microbiology results, prescriptions were made. This section is a sub-analysis of those prescriptions. In the SoC group, 179 samples were taken, accounting for 18.6% of the SoC group. In the fluorescence group, 74 samples were taken, indicating that 15.6% of the total fluorescence group was sampled.

Treatments prescribed during the same visit as the sampling was performed were statistically significantly different between the two groups (*p* < 0.0001), where oral antibiotics were the preferred medication in the SoC group, and antimicrobial dressings were the mostly used either alone or in combination with others in the fluorescence group. A detailed description is provided in Table 1.

## 4. Discussion

Antibiotic stewardship programs (ASPs) stand at the forefront of global public health initiatives to address the pervasive threat of antibiotic resistance [12,27]. These programs are indispensable in promoting judicious and responsible antibiotic use across healthcare settings and the broader community to preserve the effectiveness of antibiotics. At the same time, these initiatives foster optimal patient safety and quality of care, regulatory compliance, and adequate healthcare resources by reducing unnecessary antibiotic prescriptions. They also serve to empower healthcare professionals by providing clear guidelines that lead to evidence-based prescribing decisions; however, the scarcity of studies on antimicrobial prescription practices in the field of wound care hinders the establishment of objectively supported ASPs. This study provides a real-world depiction of current practices and shows the positive effects of implementing a bedside bacterial detection tool to reduce antibiotic use.

Fluorescence imaging is a validated tool for detecting clinically significant bacterial levels and biofilm [7]. The CIBL is the point at which wound healing is impeded by the presence of bacteria. The early detection of bacteria in nonhealing wounds allows the clinician to treat the wound locally without the use of systemic antibiotics. Moreover, the detection obviates the need for semi-quantitative cultures [8].

### 4.1. Antimicrobial and Antibiotic Prescribing

Not surprisingly, the data reported here revealed that providers working in outpatient wound centers frequently prescribe topical antimicrobials (63% of 1438 wounds). There was a notable disparity in topical antimicrobial use between sites relying solely on SoC for wound assessment and clinical decision making versus those incorporating fluorescence imaging and SoC: 87.3% of patients in the fluorescence group received topical antimicrobials compared to 50.5% in the SoC group (*p* < 0.0001). Conversely, the SoC group was 4.6 times more likely to prescribe systemic antibiotics: SoC (36.5%) versus fluorescence group (8%). The preference for topical antimicrobials as opposed to systemic antibiotics in the fluorescence group suggests that the clinicians used a more targeted approach to elevated bacterial levels. Fluorescence imaging also directs debridement to the areas of elevated bacteria levels. This has been shown by prior authors [22]. Clinicians using fluorescence imaging are more confident in treating areas of localized bacterial burden and less likely to prescribe antibiotics, particularly if the wound lacks bacterial fluorescence. Clinicians treating wounds without the aid of fluorescence imaging take a “shotgun” approach: empirically prescribing antibiotics in the hope of reducing bacterial levels that may or may not be present in the wound bed. Earlier research examining this practice concluded that it leads to the random use of antibiotics [11]. This practice is more pronounced in dark-skinned individuals [23].

The results of this study are also consistent with the recently proposed concept of biofilm-based wound care [24]. Bacteria in chronic wounds favor the biofilm phenotype. Fluorescence imaging detects both planktonic and biofilm bacteria but cannot distinguish between them [25,26]. Antibiotics are ineffective in treating CIBL or higher levels of bacteria in chronic wounds. These findings confirm that a biofilm-based treatment plan of debridement and topical antiseptics guided by fluorescence imaging is superior to relying on clinical signs and symptoms, inaccurate cultures, and empiric antibiotic therapy to treat chronic wounds. Moreover, the use of systemic antibiotics is not benign. In addition to promoting resistance, antibiotic use is associated with nosocomial infections, such as *C. difficile*, that can lead to serious and even fatal complications [1].

### 4.2. Wound Culture

Another important observation in this study pertains to the correlation between swab cultures and antibiotic prescribing. In the SoC group, 179 cultures were pending after the initial visit, representing nearly a quarter of the group. The patients were treated with empiric therapy, or the clinician waited for the microbiology results prior to initiating treatment. The use of empiric therapy leads to antibiotic overuse, while waiting for culture results delays treatment. Fluorescence imaging provides immediate results that guide antimicrobial therapy. In addition, fluorescence imaging technology can also aid in the selection of a sample site leading to more accurate microbiology results that better represent the wound’s microbiome [28]. Further research to clarify the role of fluorescence-guided polymerase chain reaction bacterial identification is ongoing.

### 4.3. The Technology

**Figure 3:**
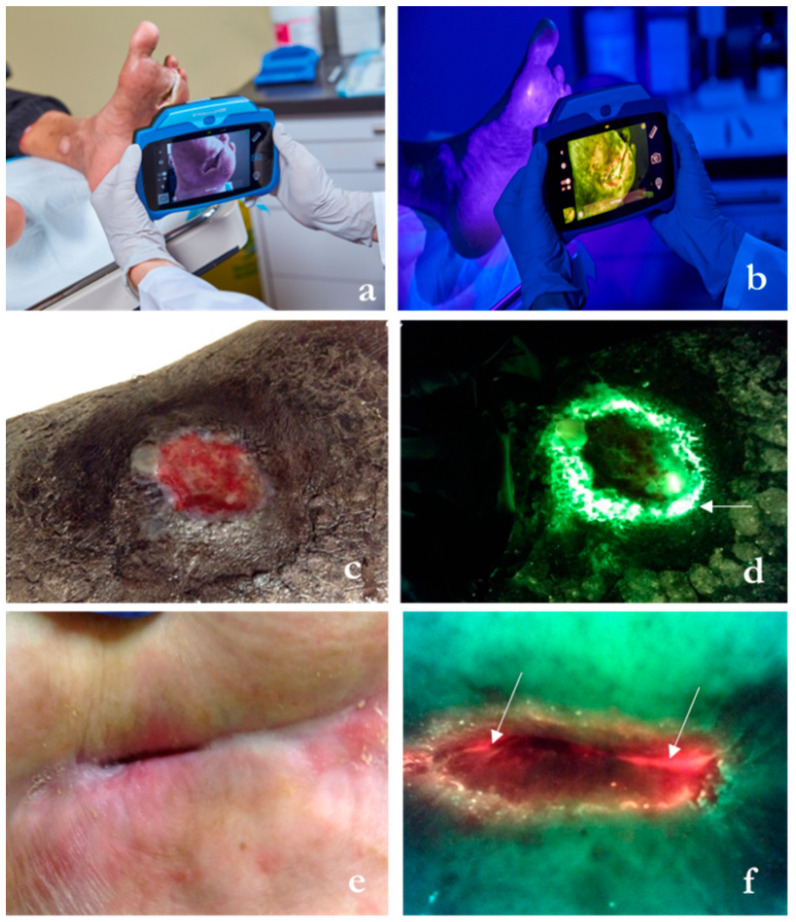
Description of the wound imaging technology employed in select wound care centers within the study, designed to bolster prescription and intervention practices directly at the patient’s bedside. (**a**) The hand-held fluorescence imaging device capturing standard images and digital wound measurement in ambient light. (**b**) Fluorescence signals are captured in darkness and appear on the screen once bacterial loads surpass 10^4^ CFU/gr of tissue, which corresponds to moderate/heavy accumulation. (**c**) Lower limb ulcer. (**d**) Corresponding fluorescence image shows cyan fluorescence (arrow), which appears as a glowing white center surrounded by a blue/green hue. A swab taken from the area of cyan fluorescence (not the wound center) detected heavy growth of *Pseudomonas aeruginosa*. (**e**) Hip pressure ulcer. (**f**) Red fluorescence indicates the presence of a multitude of gram −/+ bacterial species. This wound had a positive growth of *Staphylococcus lugdunensi* and *Providencia stuartii* at levels > 10^5^ CFU/gr of tissue (sample images courtesy of MolecuLight^®^ Inc., Toronto, ON, Canada).

**Figure 4:**
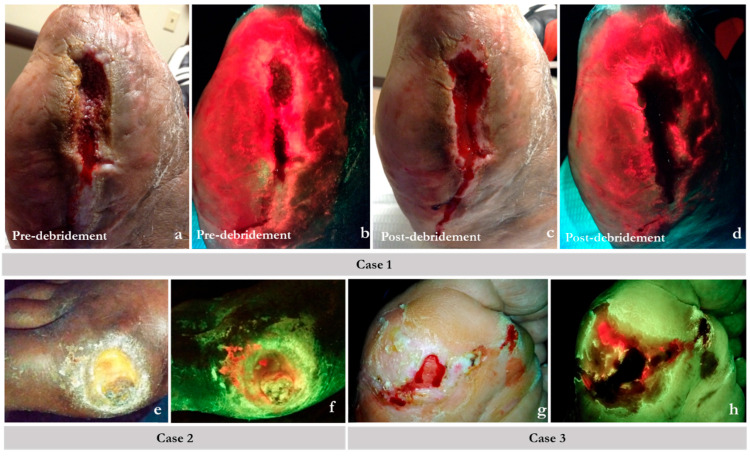
Example scenarios where fluorescence (FL) imaging guided the best course of action promoting rational use of antimicrobials. On fluorescence images, regions of red indicate chronic inhibitory bacterial loads or bacterial hotspots; normal tissue appears green. Case 1 (**a**–**d**): images showed bacterial loads persisted in a larger region than could be debrided. (**a**) Pre-cleansing/debridement standard image (ST) of a non-healing post-amputation wound site (toes were amputated on a diabetic foot). (**b**) Corresponding FL image showing extensive bacterial involvement (red) of the wound and surrounding peri-wound tissues. (**c**) Post-cleansing/debridement ST image. (**d**) Corresponding FL of the same wound showing persistence of bacterial presence (red) after debridement. The bacterial infiltration is clearly into intact skin; further debridement was not possible. In this case, an antimicrobial dressing and oral antibiotics were used. Case 2: Standard images (**e**) showing a diabetic foot ulcer (DFU). Its corresponding FL image (**f**) shows bacterial loads near the surface (red), which are best addressed topically. Systemic antibiotics are unlikely to reach this tissue in patients with poor vascularity. Case 3: (**g**) standard image of a plantar DFU post-debridement. Post-debridement FL image assessment (**h**) shows that bacterial loads could not be eliminated completely as evidenced by residual fluorescence. Bacterial fluorescence imagining objectively demonstrates the need for topical antimicrobial use.

The handheld device shown in Figure 3a identifies areas containing bacteria at concentrations greater than 10^4^ CFU/gram of tissue, either in planktonic or biofilm form, irrespective of clinical expression [19] or skin color [23]. It does so by leveraging endogenous fluorescence light emitted by certain bacterial species when exposed to violet light (Figure 3b,c). The clinician interprets the image by evaluating the fluorescence image for bacterial fluorescence. Subsequent images provide feedback on post-debridement wound hygiene. If residual fluorescence is present after initial debridement, the clinician may choose to perform further debridement or prescribe topical antiseptics (Figure 4).

### 4.4. Guidelines

Guidelines on the appropriate use of fluorescence imaging were published in 2021 using a Delphi approach [29]. The centers participating in the ASP were educated on the appropriate use of fluorescence imaging in the outpatient wound clinic. This study and further research will inform subsequent versions of the guidelines. The data presented here suggest that clinicians seeing new patients in the wound center should consider the use of fluorescence imaging.

### 4.5. Limitations

This study has limitations that warrant acknowledgment. The data, representing an initial snapshot during the patient’s first visit, offer insight into treatments administered at that moment but lack a comprehensive view of the complete patient journey. The absence of detailed information on wound severity and patients’ health and socio-economic status prevents a thorough understanding of contextual factors influencing treatment choices. While this study primarily focuses on providing an overview of prescription practices at a single time-point and how it varies in places of service that utilize fluorescence imaging, it excludes other wound treatment modalities such as cleansing and debridement. This omission should be recognized, inasmuch as the findings are confined to the examined practices related to fluorescence imaging and may not encompass the entirety of wound management strategies in clinical settings. A 2023 data collection plan is underway that will encompass these factors to provide a more comprehensive understanding of the reasoning behind prescription choices.

## 5. Conclusions

The integration of antibiotic stewardship into healthcare systems worldwide serves as a critical strategy for preserving the efficacy of antibiotics and ensuring a sustainable trajectory for global public health. Determining baseline institutional prescribing rates in addition to finding strategies that optimize the utilization of antibiotics within specific practices is a crucial first step in developing effective ASPs. In this large multicenter prospective study, the addition of fluorescence imaging to outpatient wound care centers resulted in a substantial reduction in systemic antibiotic use and an increased focus on biofilm-based practice. Fluorescence imaging plays a pivotal role in the wound clinic’s ASPs.

## Figures and Tables

**Table 1:** Prescribed treatments for SoC and Fluorescence groups.

Prescription Type	SoC	%	FL	%
Oral antibiotics	59	42.8	1	1.6
Antimicrobial dressings	26	18.8	55	87.3
Oral antibiotics and antimicrobial dressings	17	12.3	3	4.8
Topical antiseptics	8	5.8	0	0.0
Oral antibiotics and topical antibiotics	8	5.8	0	0.0
Topical antibiotics	7	5.1	0	0.0
NPWT	6	4.3	0	0.0
Oral antibiotic and topical antiseptics	2	1.4	0	0.0
Topical antibiotics and antimicrobial dressings	2	1.4	0	0.0
IV antibiotics	1	0.7	0	0.0
Oral antibiotics and antimicrobial dressing and topical antibiotics	1	0.7	0	0.0
Topical antiseptics and antimicrobial dressing	1	0.7	4	6.3

## Data Availability

The original contributions presented in the study are included in the article, further inquiries can be directed to the corresponding author.
